# The impact of relevant versus irrelevant media multitasking on academic performance during online learning: a serial of mediating models

**DOI:** 10.3389/fpsyt.2025.1599827

**Published:** 2025-08-13

**Authors:** Lifang Fan, Chen Pan, Xuejun Bai, Shiyi Li

**Affiliations:** ^1^ Faculty of Psychology, Tianjin Normal University, Tianjin, China; ^2^ School of Traffic & Transportation Engineering, Jiangxi Flight University, Nanchang, Jiangxi, China; ^3^ Key Research Base of Humanities and Social Sciences of the Ministry of Education, Academy of Psychology and Behavior, Tianjin Normal University, Tianjin, China; ^4^ Tianjin Social Science Laboratory of Students’ Mental Development and Learning, Tianjin, China

**Keywords:** academically relevant media multitasking, academically irrelevant media multitasking, self-regulation strategies, flow experience, academic performance

## Abstract

**Background:**

Online learning exhibits unique educational benefits, especially in terms of the autonomy, convenience, and flexibility it offers to students. However, it also encounters significant challenges. Without effective supervision, students are frequently distracted by checking mobile messages or other digital activities during online classes. This kind of media multitasking behavior in online learning environments has risen considerably when contrasted with traditional classroom settings. Media multitasking is typically known as simultaneously engaging in multiple media tasks or switching quickly between multiple media activities. Since task relevance impacts perceptions of multitasking and task performance, media multitasking can be categorized into academically relevant and irrelevant types. This classification enables a distinct examination of their respective correlations with academic achievement.

**Methods:**

The current study utilized a cross-sectional survey design, involving 557 Chinese university students who had participated in eight weeks of online classes. The participants completed an online survey, which incorporated the Academically Relevant Media Multitasking Questionnaire (AR-MMQ), the Academically Irrelevant Media Multitasking Questionnaire (AIR-MMQ), the Self-regulation Strategies Scale (SRS), the Flow Experience Scale (FL), and the Academic Performance Scale (AP). After conducting bivariate correlation analysis, the sequential mediation pathways were examined using structural equation modeling.

**Results:**

The findings revealed that: (1) Academically relevant media multitasking exhibited significant positive correlations with self-regulation strategies, flow experience, and academic performance. In contrast, academically irrelevant media multitasking showed no significant correlations with these variables; and (2) Self-regulation strategies and flow experience functioned as serial mediators in the relationship between academically relevant media multitasking and academic performance. However, this serial mediating effect was absent in the relationship between academically irrelevant media multitasking and academic performance.

**Conclusion:**

The findings imply that individuals who frequently participate in academically relevant media multitasking can more effectively control their behaviors, leading to enhanced concentration, a more immersive learning experience, and consequently improved academic performance. This study proposes that engaging in task-relevant media multitasking may boost cognitive processes instead of just interrupting them. It backs up a complete view where the multidimensional features of media multitasking behaviors lead to different cognitive results.

## 1 Introduction

The rapid development of mobile internet technology has fueled an information explosion, increasing students’ susceptibility to media distractions in the classroom. Studies indicate that students engage in non-course-related digital activities (e.g., messaging, social media browsing) approximately every 3–4 minutes, with each instance lasting ~1 minute (1). These behaviors result in students spending 19% - 25% of class time on such activities (2). Following the COVID-19 pandemic, online education has become integral to modern education systems, complementing traditional in-person instruction (3). While offering autonomy, convenience, and flexibility, online learning presents challenges such as ill-defined learning objectives and inadequate supervision mechanisms. Consequently, multitasking during online instruction is significantly more prevalent than in face-to-face settings (4), as learners are more easily distracted by smart devices (e.g., phones, computers) (5).

Students engaged in non-class-related media activities in class exemplify media multitasking, defined as simultaneously engaging in two or more media activities or rapidly switching among them (6, 7). Within academic environments, media multitasking occurs when students undertake academic tasks concurrently with other media activities (e.g., listening to music while reading), or switch between academic tasks and other media activities (e.g., checking messages during lectures). Consequently, such classroom media multitasking inevitably impacts students’ listening efficiency. Accumulating evidence consistently reveals a negative correlation between media multitasking behaviors and academic performance in academic contexts (8–10). On one hand, numerous correlational studies have shown that the frequency of media multitasking in either online or offline classes is significantly negatively associated with academic performance (measured by exam scores, final grades, overall GPA, self-reported comprehension of course material and overall course performance) after controlling for variables such as ACT scores, gender, attendance, and/or study time (11–13). Moreover, media multitasking not only negatively predicts concurrent academic performance but also six-month follow-up (14). On the other hand, empirical studies also confirm that students engaging in media multitasking during class exhibit significantly poorer academic performance (15), for example, Demirbilek et al. (16) discovered that students allowed to browse social websites during lectures demonstrated reduced content recall.

Prior research has focused primarily on media multitasking involving course-unrelated activities (e.g., messaging, web browsing), which disrupt primary learning tasks. However, another type of media multitasking behavior exists in the classroom. For instance, students utilize devices for course-relevant activities including accessing materials, note-taking, and participating in real-time discussions on learning platforms (17). These course-relevant activities differ fundamentally from the distractive, course-unrelated media multitasking predominantly examined in prior literature. Task relevance modulates multitasking outcomes (18, 19), with some studies showing that secondary tasks do not affect task performance when they are related to the primary task (20). Consequently, researchers have categorized media multitasking in class into on-task (academically relevant) and off-task (academically irrelevant) types, advocating for paying attention to the influence of two different types of multitasking on students (21). Only a few studies have distinguished multitasking into academically relevant and irrelevant while simultaneously exploring its effects. For example, Wood et al. (22) employed questionnaires and observational coding to track both types of media multitasking throughout an 80-minute lecture, revealing increased multitasking prevalence over time but both have no significant association with learning outcomes. Given the critical role of digital tools in education and ubiquitous device usage, it is imperative to investigate whether task-relevance driven multitasking can maintain learning efficacy or even enhance performance. Therefore, this study categorizes media multitasking in online classes into academically relevant and academically irrelevant types, aiming to elucidate the cognitive mechanisms through which each type affects academic achievement.

The critical mechanism by which media multitasking affects academic performance is probably attention. Extensive empirical evidence has established negative correlations between media multitasking and mental health, academic outcomes, and cognitive functioning, attributing these effects to attentional lapses or diminished attentional control (23, 24). Known as the attentional distraction hypothesis of media multitasking (9), this posits that habitual media multitasking impairs focused attention capacity, compromising resistance to resistance to internal (e.g., mind wandering) and external (e.g., notification alerts) distractors (6, 25). Critically, the predominant focus in the literature concerns off-task multitasking. Therefore, academically irrelevant media multitasking primarily compromises academic performance through impaired focused attention ability. Conversely, prior studies on academically relevant media multitasking have predominantly centered on students’ perspectives regarding the use of media devices for learning within the classroom (26, 27), largely neglecting its underlying cognitive mechanisms. Effective learning requires directing attention toward schema-building cognitive processes, enabling deep content processing that enhances learning outcomes (28). The Cognitive Load Theory (29, 30) posits three types of loads on working memory during learning. These include intrinsic cognitive load, which depends on the number of domain elements and their interactivity, and students’ prior knowledge or experience; extraneous cognitive load, which is evoked by the instructional design; germane cognitive load, which refers to the WM resources required to deal with intrinsic cognitive load. Intrinsic cognitive load is difficult to change, thus to facilitate effective learning, teaching should minimize extraneous cognitive load and maximize germane cognitive load. The increase in germane cognitive load depends on students’ willingness to allocate working memory resources to learning activities (31). Academically related media multitasking constitutes a strategic learning behavior for enhancing processing efficiency. Therefore, germane cognitive load may be the intrinsic cognitive process affecting the relationship between academically related media multitasking and students’ academic performance.

Self-control refers to the capacity to override or alter one’s predominant (inappropriately, impulsive, or automatic) responses to align behavior with longer-term, more rewarding goals (32, 33). It manifests in two ways: one involves the effortful suppression of short-term, gratifying impulses that hinder the achievement of long-term goals (34), and the other is proactively avoiding temptations, which actively implements self-regulation strategies (35, 36). Within the more autonomous context of online learning environments, compared with effortful impulse inhibition, actively implementing self-regulation strategies to say “no” to highly tempting stimuli may be more vital for achieving good academic results. On the one hand, Individuals who frequently engage in media multitasking (academically relevant) tend to have poorer attentional control and have difficulties in regulating their behavior (37, 38). Therefore, such individuals demonstrate infrequent deployment of self-regulation strategies. On the other hand, Self-regulation as a function of resources and perceived cognitive load (39), to increase the germane cognitive load and achieve good grades, learners will adopt effortful behaviors accordingly. Thus, frequent academically relevant media multitaskers are more likely to employ self-regulation strategies. In addition, other researchers suggested that individuals with high self-control capabilities, as compared to those with low self-control, tend to have better academic performance, and physical, and psychological well-being due to better control of attention, regulation of emotions, and suppression of impulses (40, 41). Consistent with previous studies (11), we propose Hypothesis 1: Self-regulation strategies mediate the relationship between different media multitasking types and academic performance.

Online learning requires deep immersion, specifically the psychological state termed “flow”. Flow experience, conceptualized by Csikszentmihalyi, denotes the positive emotional state occurring when individuals engage in activities with clear objectives and immediate feedback, and their skills align with the task’s challenges (42). Flow theory postulates that flow experience has nine key features: 1. Clear goals; 2. Challenge-skills balance; 3. Unambiguous feedback; 4. Sense of control; 5. Merging of action and awareness; 6. Concentration on the task; 7. Loss of self-consciousness; 8. Transformation of time; 9. Autotelic experience. Concentration on the task is a core component (43) and a prerequisite for flow experience (44, 45). On the one hand, based on the attentional distraction hypothesis of media multitasking, frequent (off-task) media multitasking has impaired focusing attention (46–48). Thus, academically irrelevant media multitasking disrupts learning tasks. Such disruptions conflict with the flow’s concentrative essence, thereby diminishing flow states (49, 50). On the other hand, there is evidence of a positive correlation between flow experiences and germane cognitive load (51). Academically relevant media multitasking, like note-taking and topic-specific searches, are effective behaviors for improving learning efficiency. It can increase the germane cognitive load (52), thereby promoting flow experiences. Additionally, other studies have found that ‘Flow implies peak performance’ (53–55). Hence, we propose Hypothesis 2: Flow experience mediates the relationship between different media multitasking types and academic performance.

**Figure 1:**
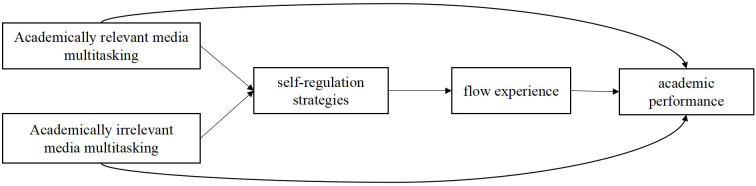
The hypothetical model.

Flow experiences facilitate learning effectiveness and foster optimal learning. Both Self-regulated learning via strategies use and flow theory emphasize the dynamic learner-environment interaction. To improve learning outcomes, learners should employ self-regulation strategies to create favorable conditions, boosting their control over the process to achieve flow (56). Specifically, the self-regulation strategies facilitates flow through the allocation of attentional resources and sustained task focus (45). Therefore, the self-regulation strategies is a precursor to flow experiences. Overall, based on the logical relationships between variables, we propose Hypothesis 3: Self-regulation strategies and flow experiences serially mediate the link between media multitasking and academic performance. The detailed path model is shown in **Figure 1**.

## 2 Methods

### 2.1 Participants and procedure

Participants were 608 undergraduate and graduate students from Tianjin Normal University. We published recruitment information in the WeChat (a popular Chinese social media platform) group in June 2022 and used the questionnaire website (https://www.wenjuan.com) to allow students to fill in questionnaires online to collect data. To expand the sample size, we adopted the snowball sampling method. We encouraged participants who saw and joined our study to share the link with more college students. To avoid data duplication, each IP address was only granted access to the survey once. The participants were told that the survey was anonymous and confidential, that the purpose of the study was to investigate media use, and that the survey was for academic purposes only. The study obtained the consent of all subjects and was approved by the Ethics Committee of Tianjin Normal University.

Due to the COVID-19 pandemic period, the school where we conducted our research was offering online teaching. Therefore, all the participants in our study underwent eight weeks of online learning. To ensure the reliability of the results of this study, we asked all participants to base their responses on what they actually did during online learning as much as possible. After excluding unqualified samples (e.g., some participants completed the questionnaire battery in < 180 s or > 15 min), we finally collected 557 valid participants with an effective response rate of 91.61%. The participants were 19.95 years old on average (M = 19.95, SD = 1.52), with 126 males (22.62%) and 431 females (77.38%).

### 2.2 Measurements

#### 2.2.1 Media multitasking questionnaire

Most of the previous studies have used the media multitasking questionnaire (MMQ) developed by Ophir et al. and calculated a media multitasking index (MMI) (6), thus MMQ can’t provide psychometric qualities. In addition, the MMQ is a complex questionnaire, that is numerous and requires participants to evaluate the time spent on each media activity, so it is not friendly for participants. In conjunction with the purpose of this study, which is to investigate the prevalence of academically relevant and academically relevant media multitasking in online classes, we drew on the media multitasking questionnaire developed by Baumgartner et al. (57). This study only required participants to answer a matrix of how often they engage in other media activities while conducting their online professional courses, and the responses were rated on a 4-point Likert scale (never = 1, occasionally = 2, often = 3, always = 4), without reporting the media use time.

The question of the academically relevant, academically relevant media multitasking questionnaire is:’To what extent do you engage in the following media activities simultaneously while working on your major courses online? Descriptions of other media activities refer to the Media multitasking questionnaire used by Madore et al. and Ophir et al. (6, 23). Academically relevant media activities included: (1) discussing class content via WeChat, Nail, QQ, etc., (2) searching or browsing for webpages or resources related to the class content, (3) reading e-books or paper books related to the course content, and (4) taking notes on paper or electronic devices; Academically irrelevant media activities included: (1) listening to music, (2) playing games, (3) watching TV, online or offline movies, (4) watching short videos (TikTok, Kwai, etc.), (5) online shopping or online transactions (using E-bank, Alipay, etc.), (6) sending and receiving messages (unrelated to classroom content) via SMS, WeChat, QQ, etc., (7) using social media (e.g., Wechat Moments, Weibo, Qzone, etc.), (8) searching or browsing webpages or resources that are not related to classroom learning, and (9) doing other things that are not related to classroom content (e.g., writing assignments for other courses, reading other books). The results of the confirmatory factor analysis (CFA) did not fully meet the criteria (the recommended model index (58): χ^2^/df < 3, CFI, TLI>0.9, RMSEA< 0.08, SRMR< 0.05), but were still within an acceptable range (the academically relevant media multitasking questionnaire: χ^2^/df=19.563/2 = 9.78, CFI=0.93, TLI=0.80, RMSEA=0.13, SRMR=0.03; the academically irrelevant media multitasking questionnaire: χ^2^/df=189.619/27 = 7.02, CFI=0.91, TLI=0.87, RMSEA=0.10, SRMR=0.05), and the academically relevant media multitasking of the internal consistency coefficient was 0.65, the Convergent Validity(AVE) was 0.34, the composite reliability (CR) was 0.66; and the academically relevant media multitasking of the internal consistency coefficient was 0.90, the convergent validity(AVE) was 0.51, the composite reliability (CR) was 0.90.

Since the aim of our study was to investigate the frequency of different types of multitasking behaviors among students during online learning, even though we made every effort to comprehensively cover all types of multitasking activities during online learning, the quantity of academically relevant multitasking behaviors is relatively limited and exhibits considerable heterogeneity. However, we believe that the above reliability and validity were acceptable in the context of our study.

#### 2.2.2 Revised online-learning motivated attention and regulation scale

The OL-MARS v.2 was developed by Wu (59) and includes two major constructs, including perceived attention problems (PAP) and self-regulation strategies (SRS). Only the SRS was used in this study, which consists of two subscales, including Behavioral Strategies (BS) and Outcome Appraisal (OA). BS measures students’ behavioral control in regulating their attention by six items. Sample questions included “When studying, I log out of my Facebook account or close instant message software so that I can focus on my work”, etc. OA measures students’ act of linking the outcome of their online learning to a specific emotion by three items. The questions included “When I notice that I am browsing unrelated sites or playing computer games, I will feel guilty” etc. The responses were rated on a 5-point Likert scale (1 = extreme disagreement, 5 = extreme agreement). The CFA revealed a good model fit (*χ* ^2^/df=4.26, CFI=0.93, TLI=0.90, RMSEA=0.08, SRMR=0.05) for the current sample, and the internal consistency coefficient was 0.81; the AVE was 0.42, CR was 0.87.

#### 2.2.3 Flow experience scale

The Flow Experience Scale in this study employed the Chinese revised version of Chang and Zhu (60), with modifications made to the prefixes of the item content to align with the context of online learning. There are four questions in this scale, e.g., “I feel time passes quickly while taking online courses”. These responses were rated on a 5-point scale (1 = disagree strongly, 5 = agree strongly). The CFA revealed a good model fit (*χ* ^2^/df = 4.13, CFI = 0.99, TLI = 0.97, RMSEA = 0.08, SRMR = 0.02) for the current sample, and the internal consistency coefficient was 0.83. the AVE was 0.56, CR was 0.84.

#### 2.2.4 Academic performance scale

Considering that this study was conducted on a large scale within the entire school during the period of online classes. Participants came from diverse academic backgrounds and followed varied curricula. Therefore, the academic performance scale developed by Long Chengzhi et al. (61) was used in this study. This scale encompasses various aspects, such as their mastery of theoretical knowledge systems, acquisition of skills in applying knowledge, and enhancement of independent thinking abilities.

Participants were asked to fill in a professional course and evaluate their subjective learning performance in it (A total of 172 different courses were listed, with 65.529% being psychology major courses. The top five courses ranked by percentage are: Educational Psychology (25.31%), Cognitive Psychology (17.59%), History of Psychology (3.95%), Psychological Statistics (3.41%), Other courses combined accounted for 46.68%). The evaluation consists of five items, e.g., “I can grasp the theoretical framework and key points of this course clearly”. The items were scored on a 5-point Likert scale (1 = disagree strongly, 5 = agree strongly). The CFA revealed a good model fit (*χ* ^2^/df = 2.51, CFI = 0.99, TLI = 0.99, RMSEA = 0.05, SRMR = 0.02) for the current sample, and the internal consistency coefficient was 0.89. the AVE was 0.63, CR was 0.89.

### 2.3 Data analysis

Data analyses were conducted using IBM SPSS 26.0 and Mplus 7.4. Firstly, descriptive analysis and correlation analysis were performed for the variables of interest for the total sample. Then Harman’s single-factor test was conducted to examine the common method bias. All of the above analyses were performed using SPSS 26.0. Subsequently, variables were centralized, and the hypothetical model was tested using Mplus 7.4. In addition, due to the complexity of the model in this study, to minimize the parameter estimation bias, we followed the recommendations of Wu and Wen’s to employ a balanced factor approach method for item parceling (62). Specifically, the dimensions of academically relevant media multitasking, the flow experience scale, and the academic performance scale, all of which have a limited number of items, were bundled into two latent variables each. Conversely, the dimensions of academically irrelevant media multitasking and the self-regulation strategies were parceled into three latent variables each, utilizing all available indicators for analysis. In the results section, for the sake of model simplicity, only the structural model is presented, excluding the measurement model.

## 3 Results

### 3.1 Common method bias analysis

Harman’s single-factor test was used to test for common method bias (63). The results of unrotated factor analysis showed that six factors with eigenvalues greater than 1 emerged, and accounted for 61.35% of the total variance. The first principal factor explained 24.81% of the variance (less than 40%). Therefore, these results indicated that common method bias was not a concern in this study.

### 3.2 Descriptive statistics and correlations

The Descriptive statistics and Pearson correlation results are shown in **Table 1**. Specifically, academically relevant media multitasking was significantly positively correlated with self-regulation strategies, flow experience, and academic performance (*r* = 0.322, *p* < 0.01; *r* = 0.400, *p* < 0.01; *r* = 0.392, *p* < 0.01), academically irrelevant media multitasking was not correlated with self-regulation strategies, flow experience, and academic performance (*r* = - 0.044, *p* = 0.297;*r* = 0.063, *p* = 0.136; *r* = - 0.008, *p* = 0.849). Since the premise of mediation analysis is that there is a significant correlation between variables, and due to the lack of significant correlations between non-academic media multitasking and other research variables, further mediation analysis will not be conducted in subsequent analyses.

**Table 1:** Mean, standard deviation, and correlation coefficient of each variable.

	M	SD	AR-MMQ	AIR-MMQ	SRS	FL
AR-MMQ	11.35	2.54				
AIR-MMQ	15.88	5.73	0.134^**^			
SRS	31.04	6.26	0.322^**^	-0.044		
FL	12.24	3.36	0.400^**^	0.063	0.501^**^	
AP	17.12	3.95	0.392^**^	-0.008	0.496^**^	0.689^**^

AR-MMQ, academically relevant media multitasking questionnaire; AIR-MMQ, academically relevant media multitasking questionnaire; SRS, self-regulation strategies scale; FL, Flow experience Scale; AP, Academic performance scale. **p < 0.01.

### 3.3 The serial mediating analysis

**Figure 2:**
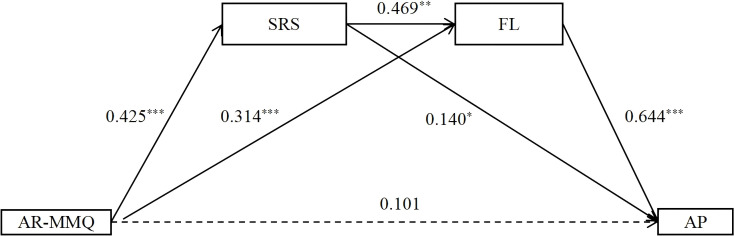
The mediating pathway of self-regulation strategies and flow experience in academically relevant media multitasking influence academical performance. AR-MMQ, Academically relevant media multitasking questionnaire; AIR-MMQ, Academically irrelevant media multitasking questionnaire; SRS, self-regulation strategies scale; FL, Flow experience Scale; AP, Academic performance scale. *p<0.05, **p<0.01, ***p<0.001.

Based on the hypothetical model, multiple mediation analysis was conducted with academically relevant media multitasking as the predictor variable, self-regulation strategies and flow experience as the mediating variables, and academic performance as the outcome variable. Additionally, age and gender were included as covariates in this chained mediation analysis. The results showed that the model fits well: *χ* ^2^/df = 2.91, CFI = 0.97, TLI = 0.96, RMSEA = 0.06, SRMR = 0.04. The detailed path model is shown in **Figure 2**. Specifically, academically relevant media multitasking couldn’t significantly predict academic performance (β *=* 0.101, *p =* 0.068), but it could positively significantly predict self-regulation strategies and flow experience (β *=* 0.425, *p <* 0.001; β *=* 0.314, *p <* 0.001). In addition, self-regulation strategies positively significantly predicted flow experience, and academic performance (β *=* 0.469, *p <* 0.001; β *=* 0.140, *p <* 0.05), and flow experience also positively significantly predicted academic performance (β *=* 0.644*, p <* 0.001).

Then, we performed a bootstrap analysis using the bias correction non-parametric percentage test to further examine the serial mediating effects. The results revealed that the direct effect of academically relevant media multitasking on academical performance was not significant (*p* = 0.068). the self-regulation strategies and flow experience were found to play a mediating role in the relationship between academically relevant media multitasking and academic performance. Specifically, this mediating effect consists of three pathways (see **Table 2**), indirect pathway 1: academically relevant media multitasking →self-regulation strategies → academic performance; indirect pathway 2: academically relevant media multitasking→ flow experience → academic performance; indirect pathway 3: academically relevant media multitasking →self-regulation strategies →flow experience → academic performance. The effect values of the three pathways were 12.2%, 41.1%, and 26.1%, respectively. The 95% confidence intervals of the three paths did not contain 0, indicating that the serial mediation effect was significant.

**Table 2:** Mediating paths between academically relevant media multitasking and academical performance.

	Effect	Boot SE	Boot LLCI	Boot ULCI	Ratio (%)
Total	0.591				
Total indirect effect	0.469	0.080	0.330	0.650	
Indirect effect 1	0.072	0.035	0.015	0.159	0.122
Indirect effect 2	0.243	0.070	0.128	0.404	0.411
Indirect effect 3	0.154	0.034	0.099	0.237	0.261

Relative effect (%) = Indirect effect/Total;Indirect pathway 1: academically relevant media multitasking →self-regulation strategies → academic performance; Indirect pathway 2: academically relevant media multitasking→ flow experience → academic performance; Indirect pathway 3: academically relevant media multitasking →self-regulation strategies →flow experience → academic performance.

## 4 Discussion

First, this research revealed a significant positive correlation between academically relevant media multitasking and academic performance, aligning with prior studies (64). For example, in a study conducted by Kuznekoff et al., participants were instructed to take notes while viewing courseware videos. Results indicated that the experimental group engaged in messaging exhibited inferior note quality and poorer recall test performance compared to the control group. Nevertheless, when further dividing the experimental group based on message content relevance to the courseware, the relevant subgroup demonstrated notably better note quality and recall performance than the irrelevant subgroup, with no significant difference from the control group (65). These findings suggest that the effects of media multitasking on academic performance stem not from multitasking per se but from the purpose behind media usage.

Second, this study confirmed that academically relevant media multitasking significantly and positively predicted self-regulation strategies scores, contrasting with prior reports of self-control deficits among frequent multitaskers. Self-control comprises effortful impulse inhibition and self-regulation strategies activation (66–68). Prior research mostly involved media multitasking unrelated to main tasks. Consistent with the attentional distraction hypothesis of media multitasking, frequent engagement in such behaviors reduced attentional control and impulse inhibition, thereby impairing self-control. This primarily affects effortful inhibition rather than self-regulation strategies use, accounting for the non-significant association between academically irrelevant media multitasking and self-regulation strategies in this study. Conversely, academically relevant media multitasking (e.g., note-taking, course-related searches) represents both a performance-enhancing strategies and a volitional attempt to optimize germane cognitive load for improved learning. Furthermore, students frequently multitasking for learning purposes may be better able to make adaptive use of media devices, such as utilizing the adaptable commitment device (69), making it clear to the device what you want (e.g. entertainment or focus) so that the device can show it different content. This aligns with the use of self-regulation strategies to achieve set goals. Collectively, these mechanisms explain the positive academically relevant media multitasking → self-regulation linkage. Consistent with previous findings (70), self-regulation strategies positively predict individual academic performance. Thus, H1 receives partial support: self-regulation mediates specifically the academically relevant media multitasking → performance pathway.

Third, this study also partially confirmed Hypothesis 2, revealing that flow experience mediates the relationship between academically relevant media multitasking and academic performance. Flow theory positions focused attention as its core prerequisite and defining characteristic (71). Although prior research shows a negative correlation between media multitasking and focusing attention, this study found that academically relevant media multitasking positively predicted flow experience. This may be because academically relevant multitasking does not distract from the learning task. Furthermore, task coherence facilitates information integration, enabling cognitive resource reallocation toward germane load processing (72). Critically, greater task correlation reduces perceived cognitive demands, promoting skill-challenge balance that enhances flow experience (73). As flow positively predicts academic performance (54), this confirms its mediating role in the relationship between academically relevant media multitasking and academic performance.

Finally, self-regulation strategies and flow experience play serially mediating roles in the relationship between academically relevant media multitasking and academic performance. Consistent with previous research, elevated self-control predicts enhanced flow states (74). During academically relevant media multitasking activities, such as taking notes and consulting course materials on media devices, learners’ primary aim is learning outcome optimization. Consequently, they employ various self-control measures to resist distractions and maintain focus on their learning tasks. T Focused attention constitutes a prerequisite for flow (75). Therefore, Hypothesis 3 is validated.

Notably, this study found that the academically irrelevant media multitasking demonstrated a non-significant association with academic performance. Predominant literature conceptualizes academically irrelevant multitasking as “cyberslacking”, presuming it invariably compromises academic outcomes through distraction. However, there was also congruent evidence exists in the present study (76). It was also found that having participants reply to messages while reading did not negatively affect comprehension of the text content, but merely increased reading time (77). Scholars attribute this to self-control moderation. High-self-control individuals detect task interference upon task-switching and compensate via strategic re-engagement (e.g., content review). Thus, compensatory strategies (e.g., re-reading) maintain comprehension despite temporal costs (78). Therefore, the above research findings suggest that the relationship between academically irrelevant media multitasking and academic performance is moderated by self-control and confounded by methodological factors (e.g., assessment content, and evaluation metrics).

## 5 Limitation and prospects

The advancement of mobile communication technology and the explosive growth of information in the smart era frequently distract individuals with multiple information streams, rendering media multitasking a prevalent behavior. We must acclimate to this “new normal” and evaluate its implications. To unpack these consequences, this study innovatively classifies media multitasking behaviors in online classrooms into two types based on task relevance: 1. Academically Relevant Media Multitasking: secondary tasks that directly support the class content (e.g., material retrieval, collaborative discussions); and 2. Academically Irrelevant Media Multitasking: secondary tasks that distract from class content (e.g., social networking, entertainment). Notably, fundamentally distinct impact pathways were identified: academically relevant media multitasking indirectly enhanced academic performance by strengthening self-regulation strategies and flow experience. In contrast, academically irrelevant media multitasking demonstrated no significant association with academic performance. These findings partially corroborate cognitive load theory and provide critical insights for leveraging media multitasking to improve learning performance. For instance, by employing the “Content modification” approach in media usage interventions (79), such as modifying browser search bars to make learning-related content more prominent or using browser extensions to remove irrelevant news feeds, so that converting the “multitasking norm” into a strategic tool that enhance germane cognitive load and, consequently, learning performance.

Several limitations warrant attention. First, academically relevant media multitasking operationalization relied on subjective frequency reports of classroom-relevant media behaviors without accounting for functional heterogeneity within subtypes (e.g., material retrieval/real-time discussions/digital note-taking). Critically, even goal-relevant behaviors (e.g., cross-website material retrieval) may induce extraneous cognitive load through interface switching, exposure to distractive information, or task shifting, potentially offsetting benefits. Future studies should disaggregate academically relevant media multitasking into subtypes (e.g., integrated-platform searches vs. open-web searches) and utilize eye-tracking and cognitive-load scales to model the dynamic interplay between germane, as well as extraneous load and their joint influence on achievement. Second, data collection during the COVID-19 pandemic restricted us to self-report questionnaires, which inadequately capture complex, transient, and context-sensitive constructs. Therefore, future research could incorporate other methods, such as diary studies and intensive longitudinal designs, to construct multimodal behavioral datasets and obtain more authentic and accurate patterns of learner behavior. Third, measuring academic performance via self-evaluation rather than objective grades (due to privacy constraints and grading heterogeneity) limits cross-study comparability with objectively measured outcomes. Future studies should integrate standardized objective metrics. Fourth, this study is a cross-sectional study. Although it identified a pathway through which academically relevant media multitasking promotes self-regulation strategies and flow experience, thereby enhancing academic performance, it remained difficult to draw definitive conclusions about the causal order among the variables. Future research should adopt longitudinal and experimental approaches to investigate the relationships between media multitasking and academic performance, along with possible mediating or moderating factors, to reinforce and broaden the research outcomes.

## 6 In conclusion

Our study found that self-regulation strategies and flow experience acted as serial mediators in the relationship between academically relevant media multitasking and academic performance. The findings suggest that individuals who frequently engage in academically relevant media multitasking are better able to take effective measures to regulate their multitasking behaviors, resulting in more focused attention, a more immersive flow experience, and therefore better academic performance.

## Data Availability

The original contributions presented in the study are included in the article/supplementary material, further inquiries can be directed to the corresponding author/s.
