# Correction: The impact of relevant versus irrelevant media multitasking on academic performance during online learning: a serial of mediating models

**DOI:** 10.3389/fpsyt.2025.1685646

**Published:** 2025-08-27

**Authors:** Lifang Fan, Chen Pan, Xuejun Bai, Shiyi Li

**Affiliations:** ^1^ Faculty of Psychology, Tianjin Normal University, Tianjin, China; ^2^ School of Traffic & Transportation Engineering, Jiangxi Flight University, Nanchang, Jiangxi, China; ^3^ Key Research Base of Humanities and Social Sciences of the Ministry of Education, Academy of Psychology and Behavior, Tianjin Normal University, Tianjin, China; ^4^ Tianjin Social Science Laboratory of Students’ Mental Development and Learning, Tianjin, China

**Keywords:** academically relevant media multitasking, academically irrelevant media multitasking, self-regulation strategies, flow experience, academic performance

In the published article, there was an error in the **Funding** statement. During manuscript preparation, a grant number was inadvertently copied from another document and pasted into the **Funding** section, resulting in the inclusion of an entirely unrelated project number. The **Funding** originally read: “The author(s) declare financial support was received for the research and/or publication of this article. This study was supported by the National Natural Science Foundation of China (31800921).”

The correct **Funding** statement appears below.

“The author(s) declare financial support was received for the research and/or publication of this article. This study was supported by Tianjin Philosophy and Social Science Planning project (TJJX22-007).”

The original article has been updated.

